# A Computational Framework to Characterize the Cancer Drug Induced Effect on Aging Using Transcriptomic Data

**DOI:** 10.3389/fphar.2022.906429

**Published:** 2022-06-29

**Authors:** Yueshan Zhao, Yue Wang, Da Yang, Kangho Suh, Min Zhang

**Affiliations:** ^1^ Center for Pharmacogenetics, Department of Pharmaceutical Sciences, University of Pittsburgh, Pittsburgh, PA, United States; ^2^ UPMC Hillman Cancer Institute, University of Pittsburgh, Pittsburgh, PA, United States; ^3^ Department of Computational and Systems Biology, University of Pittsburgh, Pittsburgh, PA, United States; ^4^ Department of Pharmacy and Therapeutics, University of Pittsburgh, Pittsburgh, PA, United States

**Keywords:** transcriptomics, cancer drug, aging, pharmacogenomics, drug-aging interaction

## Abstract

Cancer treatments such as chemotherapies may change or accelerate aging trajectories in cancer patients. Emerging evidence has shown that “omics” data can be used to study molecular changes of the aging process. Here, we integrated the drug-induced and normal aging transcriptomic data to computationally characterize the potential cancer drug-induced aging process in patients. Our analyses demonstrated that the aging-associated gene expression in the GTEx dataset can recapitulate the well-established aging hallmarks. We next characterized the drug-induced transcriptomic changes of 28 FDA approved cancer drugs in brain, kidney, muscle, and adipose tissues. Further drug-aging interaction analysis identified 34 potential drug regulated aging events. Those events include aging accelerating effects of vandetanib (Caprelsa®) and dasatinib (Sprycel®) in brain and muscle, respectively. Our result also demonstrated aging protective effect of vorinostat (Zolinza®), everolimus (Afinitor®), and bosutinib (Bosulif®) in brain.

## 1 Introduction

Cancer survival has been significantly improved because of better screen/diagnosis strategies and more effective treatments. The number of cancer survivors is projected to increase to 22.2 million by 2030 in the United States. However, the cancer survivors are at increased risk for accelerated aging and related health conditions (Guida et al., 2019). The normal aging process is described as a gradual accumulation of molecular and cellular damage, which eventually leads to systematic dysregulation. Cancer treatments such as chemotherapies can also cause genotoxic and cytotoxic damage to normal tissues in the cancer patients. This may change or accelerate the aging process in multiple organs of patients in long term. Emerging clinical studies have shown that cancer treatments can lead to a broad spectrum of aging-related health conditions at a younger age for the cancer survivor (Guida et al., 2021).

Clinical follow-up studies indicate that cancer treatment contributes to the early onset of aging-related symptoms in the young cancer survivors, such as frailty, incident comorbidities, functional loss, and cognitive decline (Guida et al., 2019; Guida et al., 2021). Furthermore, observational studies have shown that survivors of adult-onset cancers have a higher burden of mobility limitations (Keating et al., 2005), comorbid conditions (Alfano et al., 2017), pain (Alfano et al., 2017), and a greater risk of functional and cognitive impairments compared with healthy, age-matched controls (Hewitt et al., 2003). These preclinical and clinical findings suggest that cancer treatments may lead to a change or acceleration of the aging trajectory. Several cancer drugs have been shown to cause cell damage through mechanisms similar to those mediating the aging process (Guida et al., 2019). However, for most of the FDA approved cancer drugs, the molecular and cellular changes underlying the interaction of cancer treatment and altered aging trajectory are unknown. This limitation has constrained the efforts to identify, predict, and mitigate the aging-related consequences for cancer survivors (Guida et al., 2021).

Emerging evidence has shown that transcriptome and other types of “omics” data can be used to study molecular changes and trajectory of the aging process (Edwards et al., 2007; Peters et al., 2015). For example, by studying the epigenome and transcriptome landscapes of mice in different age groups, Benayoun and collogues have revealed widespread induction of inflammatory responses during the aging process (Benayoun et al., 2019). Omics data analyses have shown that under-expression of mitochondrial gene in tissues from aged donors (Yang et al., 2016). Study in transcriptomes across multiple species with varied lifespans demonstrated feasibility of using gene expression analysis to characterize the molecular signatures of longevity (Ma et al., 2018). DNA-methylation aging markers have been identified using the epigenetic profile as a “clock” of aging (Horvath and Raj, 2018). Despite recent development in transcriptomic and epigenetic research in the normal aging process, limited work has been done to characterize the cancer drug-induced aging process in cancer survivors.

This gap of knowledge is in part because of the paucity of samples and data that can be obtained after cancer treatment. The LINCS L1000 (L1000) transcriptome database is a comprehensive gene expression knowledgebase of pre- and post-treatment cell lines (Subramanian et al., 2017). This collection of post-treated expression profiles is an important resource for finding the connections between drugs, therapeutic effects, and disease states. In addition to the L1000 data, the availability of the multiple normal tissue transcriptomic data from the Genotype-Tissue Expression (GTEx) database (Consortium et al., 2017) allows us to robustly characterize an aging-associated signature in each tissue. In this regard, we hypothesize that we can integrate the L1000 and GTEx databases to investigate the scope and molecular changes of cancer drug-induced aging processes by comparing the transcriptional profiles of normal aging tissues and post-treatment normal cell lines. To test this hypothesis, we have developed a computational framework to identify aging-associated signatures from GTEx, the drug-induced expression signatures from L1000 database, and further compared these two signatures to study the drug-aging interaction and underlying molecular changes during this process. Among the significant drug-aging interactions are aging-accelerating effect of vandetanib in brain and dasatinib in muscle. Meanwhile, the protective effect of vorinostat and bosutinib on brain aging, and everolimus’ different roles in muscle and brain aging processes are characterized. Our integrative study successfully characterized the drug-aging interaction maps of 28 FDA approved cancer drugs in four normal tissues.

## 2 Materials and Methods

### 2.1 Expression Data

To date, the L1000 database (Subramanian et al., 2017) has included more than 1.3 million post-treated expression profiles from both normal and cancer cell lines. The majority of these gene expression profiles comprise transcriptional responses of human cancer cells to chemical and genetic perturbations. Phase II L1000 data of the best inferred gene space were obtained from https://lincsproject.org/LINCS/.

The Genotype-Tissue Expression (GTEx) database (Consortium et al., 2017) contains gene expression profiles from multiple tissue sites across nearly 1,000 individuals. Version 8 (V8) data for 46 tissue types with at least 84 samples for following analysis were downloaded from GTEx Portal (https://www.gtexportal.org/).

### 2.2 Statistical Analysis

Spearman correlation coefficient (Schober et al., 2018) between each gene’s expression and the age of the corresponding donors in each tissue type were calculated. The *p*-value <0.05 was used to select significant correlations. Gene Set Enrichment Analysis (GSEA) (Subramanian et al., 2005) were performed on the genes ranked based on their correlation with donor ages. Gene Ontology (Ashburner et al., 2000; Wu et al., 2020), KEGG (Kanehisa and Goto, 2000), and curated gene sets from the Molecular Signatures Database (MSigDB) (Liberzon et al., 2015) were used to identify the pathways that enriched with aging/treatment-associated genes. All the computational and statistical analyses were performed using R (version 3.6.2) and Python (version 3.8.0).

### 2.3 Identification of Aging-Associated and Drug-Induced Signatures

In the GTEx dataset, Spearman correlation analysis was performed to identify the gene expression that was significantly correlated with the ages of the donors in different tissue types respectively. In each tissue, genes with *p*-values <0.05 and Spearman correlation coefficient rho> 0.2/< −0.2 were selected as aging-associated signature genes. In some tissue types, we found very few aging-associated genes using the above criterion which might be caused by the small sample size. Then we chose 500 genes with top positive correlation and 500 genes with top negative correlation with donor ages as the signature genes for the following study. The signature *r* contains signature genes with positive correlation assigned with “+1,” signature genes with negative correlation assigned with “−1,” and the rest assigned with “0”.

In the L1000 dataset, drug-induced signatures were built on genes that significantly changed after treatment using experiments with high reproducibility among replicates. For the transcriptomes treated with the same drug, genes that have significant changes with z score >1 or < −1 in 20% or more experiments were selected as signature genes similar to previously described in (Stathias et al., 2018). After drug treatment in the normal cells, each drug-induced signature *z* includes significantly upregulated genes assigned with “+1,” significantly downregulated genes assigned with “−1,” and the rest of the genes marked as “0”.

### 2.4 Integrating the Drug-Induced Signatures and Aging-Associated Signatures to Identify Cancer Drug-Aging Interactions

**FIGURE 1:**
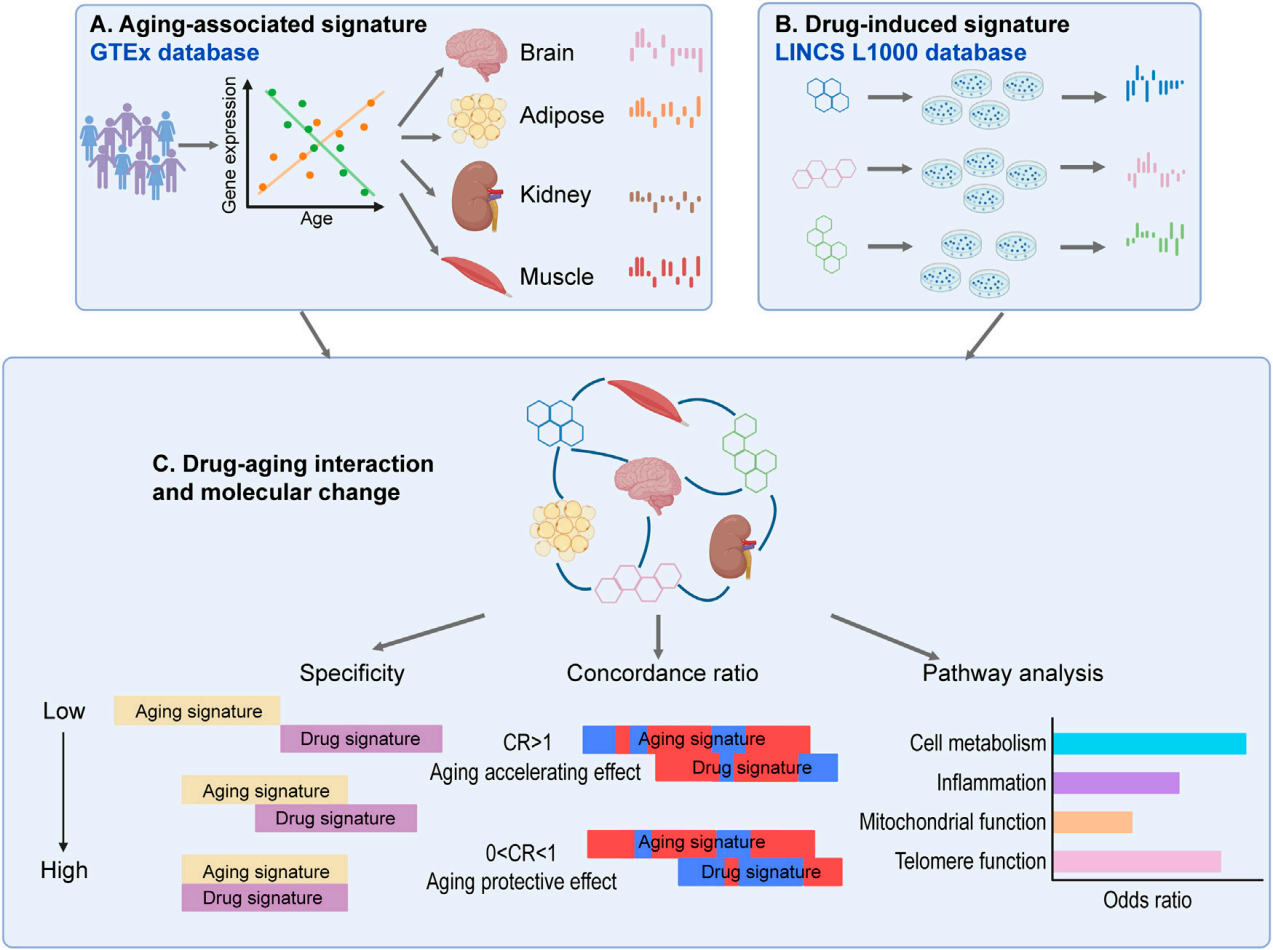
The computational framework to characterize the cancer drug-aging interaction. **(A)** Identification of aging-associated expression signatures in different tissue types in GTEx database. **(B)** Identification of drug-induced expression signatures in different normal cells from LINCS L1000 database. **(C)** Interaction of drug-induced signature and aging-associated signature characterized by specificity, adjusted concordance ratio and enriched functional pathways.

9,792 overlapped genes between the LINCS L1000 database and GTEx data were used to investigate the interaction between drug-induced signatures and aging-associated signatures as shown in Figure 1. The specificity (S) score and concordance ratio (CR) similar to (Stathias et al., 2018) were used to quantify the signature interaction and evaluate the drug-induced aging effects. The specificity score was calculated as the percentage of shared genes between the drug-induced signature in a specific cell line and the aging-associated signature in a matched tissue type comparing to the total number of genes in the aging-associated signature in the matched tissue type (Eq. 1). The range of the specificity score is between 0 and 1. Higher specificity score indicates higher overlap between two signatures.

$$Specificity=\;\frac{\sum_{i=1}^{9792}\left[a_{i}\cdot b_{i}\right]}{\sum_{i=1}^{9792}\left[a_{i}\right]}\;with\;a_{i}=\{\begin{array}{c} 1,\;if\;r_{i}\neq 0\\ 0,\;if\;r_{i}=0 \end{array}\;and\;b_{i}=\{\begin{array}{c} 1,\;if\;z_{i}\neq 0\\ 0,\;if\;z_{i}=0 \end{array}\;\;$$ (1)

The concordance ratio was defined as the ratio of the number of shared genes in both signatures that are upregulated/downregulated after drug treatment and positively/negatively correlated with increased donor ages versus the number of shared genes in both signatures that are upregulated/downregulated after drug treatment but negatively/positively correlated with increased donor ages (Eq. 2).

$$Concordance\;ratio=\frac{\sum_{i=1}^{9792}\left[a_{i}\right]}{\sum_{i=1}^{9792}\left[b_{i}\right]}\;with\;a_{i\;}=\{\begin{array}{c} 1,\;if\;z_{i}\cdot r_{i}>0\\ 0,\;if\;z_{i}\cdot r_{i}<0 \end{array}\;and\;b_{i}=\{\begin{array}{c} 1,\;if\;z_{i}\cdot r_{i}<0\\ 0,\;if\;z_{i}\cdot r_{i}>0 \end{array}\;$$ (2)

where *z* and *r* are the vectors of drug-induced signature and aging-associated signature respectively. An adjusted concordance ratio is further obtained by adding a pseudocount of 1 to both of numerator and denominator of the original ratio, which can be used to characterize the cases with denominator at zero when calculating the concordance ratio. For example, when no overlapped genes or no genes with opposite alteration direction were found between aging-associated signature and drug-induced signature, adjusted concordance ratio will provide a proper calculation. Moreover, a permutation test will be performed for each adjusted concordance ratio to show its significance. In each permutation, an adjusted concordance ratio will be calculated for the randomly selected aging-associated signature and drug-induced signature keeping the number of positive and negative genes remains the same with the original signatures. This process will be repeated for 10,000 permutations and a normal distribution is approximated for permutated adjusted concordance ratios, which will be used to calculate the *p*-value for the observed adjusted concordance ratio. A significant adjusted concordance ratio <1 suggests a potential protective effect from aging, and a significant concordance ratio >1 indicates more likely the drug can accelerate aging-related process in this specific tissue. Further enrichment analysis was performed on genes that were shared between aging-associated signature and drug-induced signature, using pathway annotations from Gene Ontology (GO) (Gene Ontology, 2004) by Enrichr (Chen et al., 2013) with Fisher’s exact test.

## 3 Results

### 3.1 Aging-Associated Gene Expression in the GTEx can Recapitulate the Well-Established Aging Hallmarks

**FIGURE 2:**
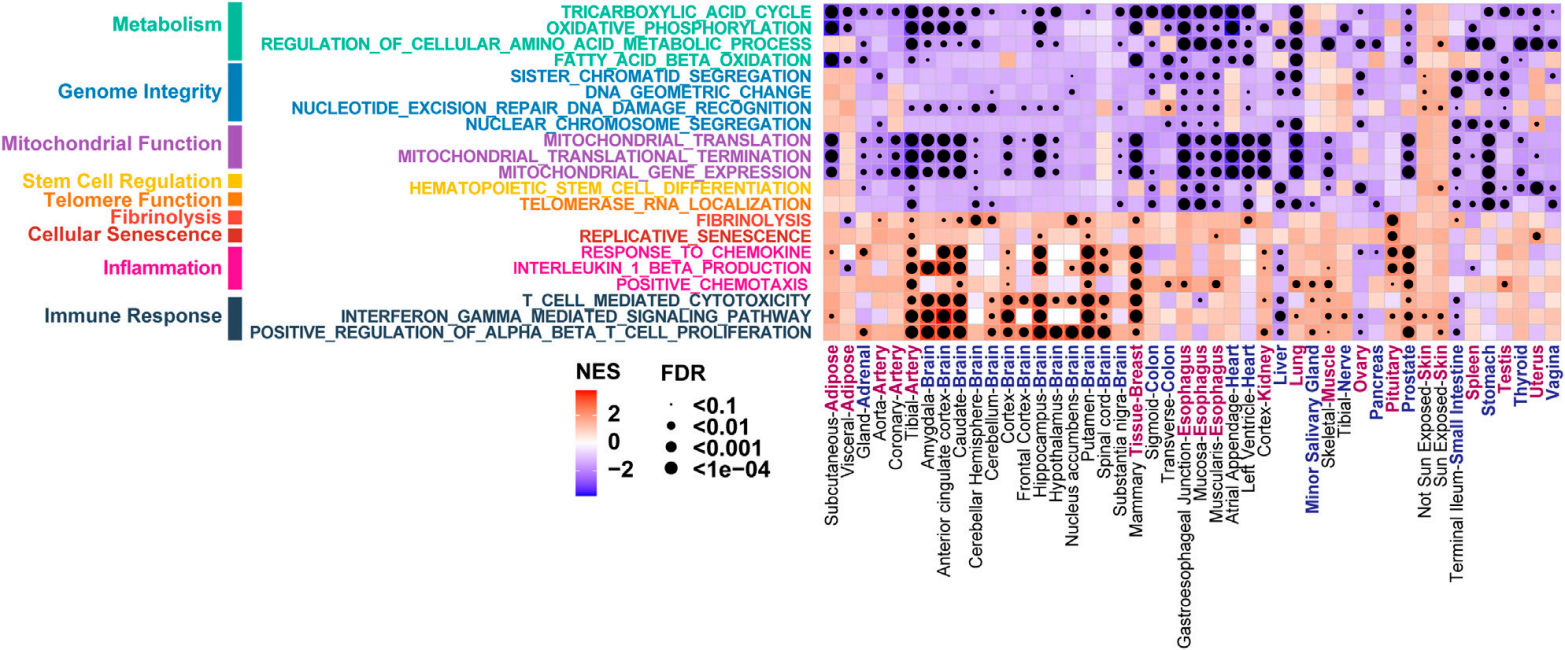
Aging-associated gene expression in the GTEx can recapitulate the well-established aging hallmarks. The Normalized Enrichment Score (NES) and False Discovery Rate (FDR) of GSEA analysis are shown for gene sets grouped by different aging hallmarks in each tissue type.

The GTEx database contains gene expression profiles from multiple tissue sites across nearly 1,000 individuals aged from 20 to 71. We first sought to determine if gene expression data can be used to characterize the molecular changes during the normal aging trajectory. For each of 46 tissue types that has at least 84 samples in the GTEx database (V8), we performed GSEA analyses on the genes ranked based on their expression correlation with donor ages. This analysis revealed that the aging-associated transcriptomic changes are highly consistent with established aging hallmarks (Lopez-Otin et al., 2013). As shown in Figure 2, significant positive correlations were observed between the increased donor ages and increased gene expression in cell senescence (REPLICATIVE SENESCENCE, FDR <0.1 in 6 out of 46 tissue types) and inflammation (POSITIVE CHEMOTAXIS, FDR <0.1 in 14 out of 46 tissue types). In addition to the activated cellular process, the decline of some molecular and cellular functions is also prominent as donor age increases. For example, the increased donor ages are highly correlated with decreased gene expressions in telomere function (TELOMERASE RNA LOCALIZATION, FDR <0.1 in 18 out of 46 tissue types), mitochondrial function (MITOCHONDRIAL GENE EXPRESSION, FDR <0.1 in 27 out of 46 tissue types), and genome integrity (NUCLEAR CHROMOSOME SEGREGATION, FDR <0.1 in 12 out of 46 tissue types) (Figure 2). These results suggest the aging process in multiple tissue types can be characterized by the gene expression profiles.

**FIGURE 3:**
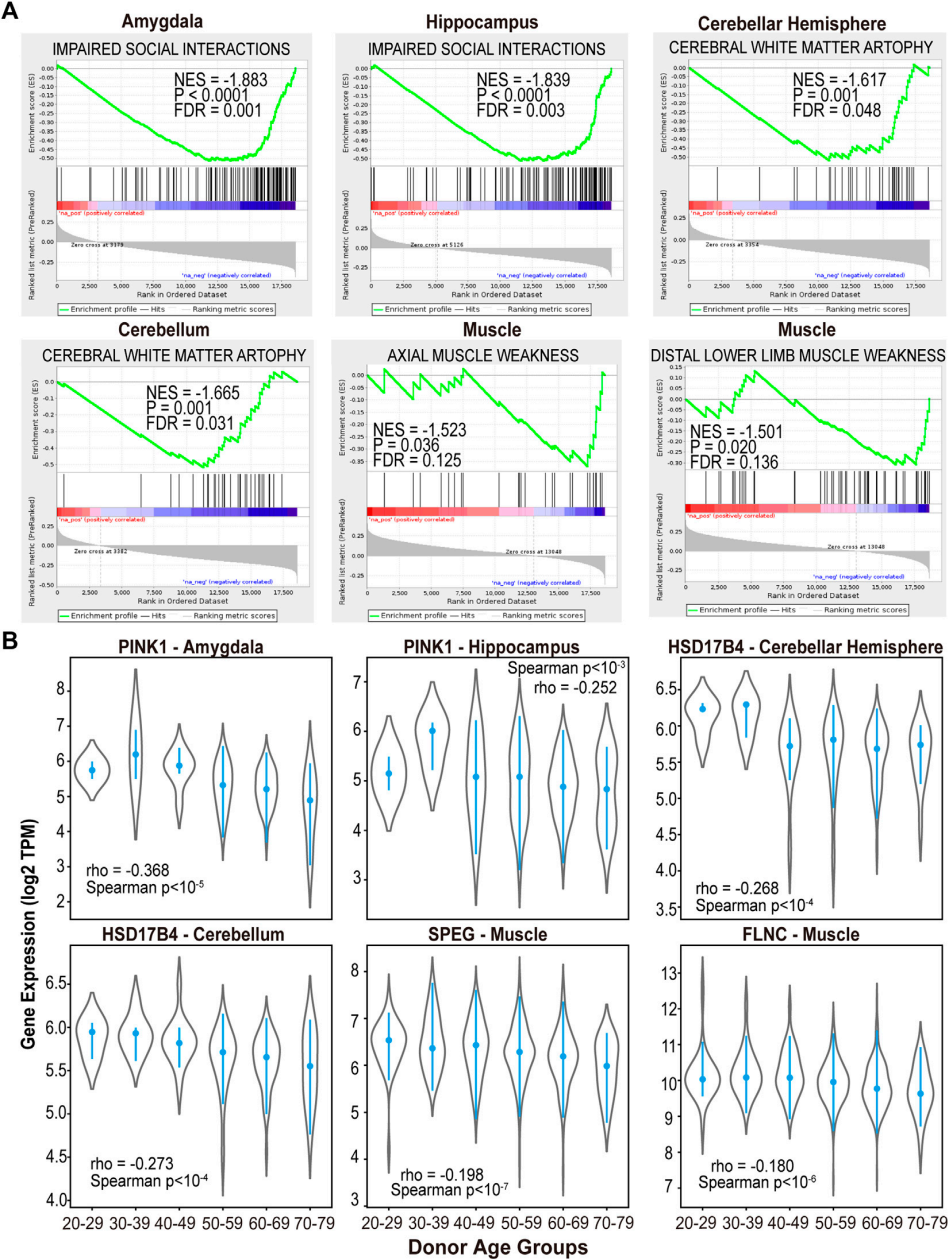
The aging-associated genes in normal brain and muscle tissues are highly overlapped with brain and muscle aging phenotypes. **(A)** GSEA analysis of aging-associated genes using gene sets derived from the Human Phenotype Ontology. **(B)** Decreased gene expression in normal brain and muscle tissues of aged donors.

In the amygdala and hippocampus regions of the brain, the aging-associated genes are highly overlapped with characteristics associated with neurodegeneration phenotypes such as “IMPAIRED SOCIAL INTERACTIONS” (Figure 3A). Specifically, PTEN induced kinase 1 (PINK1) is one of the core genes that are significantly decreased in aged donors in the IMPAIRED SOCIAL INTERACTIONS function (Figure 3B). Previous studies have shown that PINK1 plays a vital role in mitochondrial maintenance (Wilhelmus et al., 2011), and PINK1 dysregulations may contribute to neurodegeneration and aging (Kitagishi et al., 2017), which is consistent with our results here. Dysfunction of PINK1 related pathway has been reported in patients with Parkinson’s and Alzheimer’s diseases (Ye et al., 2015; Martin-Maestro et al., 2016), which makes it a candidate therapeutic target for those diseases (Du et al., 2017).

Meanwhile, neurodegeneration associated function “CEREBRAL WHITE MATTER ATROPHY” (Patel and Barkovich, 2002; Poretti and Boltshauser, 2015) demonstrated enrichment with genes that significantly decreased in aged donors’ cerebellum and cerebellar hemisphere of brain (FDR <0.05, Figure 3A). Among 29 core genes in this functional module, Hydroxysteroid 17-Beta Dehydrogenase 4 (HSD17B4) showed significantly low expression as the donor’s age increases (Spearman *p* < 10^−4^, Figure 3B). HSD17B4 has been reported to play an important role in the catalyzing β oxidation of very long chain fatty acids (VLCFA) (Violante et al., 2019), and mutations in this gene can cause progressive cerebral atrophy in clinical cases (Amor et al., 2016; Landau et al., 2020).

In the muscle skeleton samples, the significantly downregulated genes in aged donors were enriched in functions of “AXIAL MUSCLE WEAKNESS” and “DISTAL LOWER LIMB MUSCLE WEAKNESS” (FDR <0.15, Figure 3A). For example, striated preferentially expressed gene (SPEG) and Filamin C (FLNC), involved in the two pathways respectively, showed significantly decreased expression in muscle samples of aged donors (Spearman *p* < 10^−7^ and *p* < 10^−6^, Figure 3B). Consistent with our results, SPEG deficiency in skeletal muscle was found to cause defective calcium handling and excitation-contraction coupling, further lead to congenital myopathies (Huntoon et al., 2018). Meanwhile, previously research revealed a clinical case with FLNC mutations experienced weakness in the lower limbs and proximal muscles (Chen et al., 2019). Moreover, previous study has shown that genetic variants in FLNC is one of the prevalent causes of myopathies and cardiomyopathies (Verdonschot et al., 2020). Our results suggest muscle weakness, the main phenotype of aging process in skeletal muscle, can be characterized by the transcriptome profiles from donors of different ages.

### 3.2 Cancer Drug Induced Transcriptomic Changes Are Highly Resembled to Normal Aging Processes in Multiple Tissue Types

**FIGURE 4:**
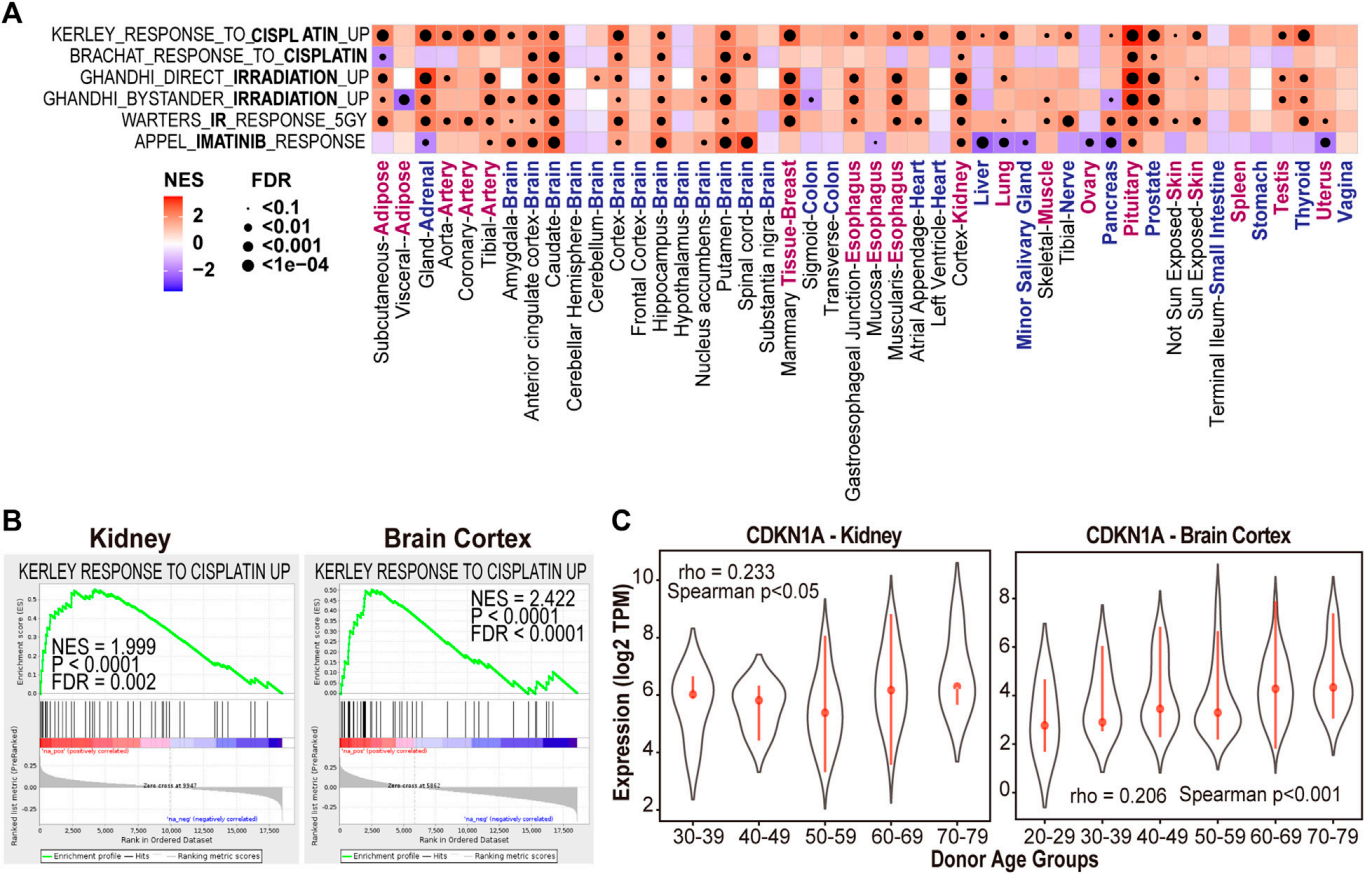
Aging-associated genes are enriched with cancer drugs induced gene sets in multiple tissue types. **(A)** NES and FDR of GSEA analysis of treatment-induced gene sets in each tissue type. **(B)** Cisplatin induced gene set enriched with genes highly expressed in kidney and brain cortex samples of aged donors. **(C)** CDKN1A expression in kidney and brain cortex tissues of different donor age groups.

In addition to the molecular and cellular processes, we also investigated if aging-associated genes in multiple tissue types can enrich previously identified cancer drug-induced pathways. The GSEA analysis revealed that cisplatin induced genes are highly enriched in the overexpressed genes in 27 out of 46 tissue types of the aged donors, including multiple regions of brain, adipose, muscle and kidney (FDR <0.1, Figure 4A). For example, genes with positive expression correlation with increased donor ages in the brain cortex region are highly overlapped with the core genes that are induced after cisplatin treatment in cancer cells (KERLEY RESPONSE TO CISPLATIN UP (Kerley-Hamilton et al., 2005), FDR <0.1, Figure 4B). In particular, cyclin dependent kinase inhibitor 1A (CDKN1A) is one of shared genes exhibited significant over-expression as the donor’s age increase in kidney and brain cortex region (Figure 4C). This observation is consistent with previous report of CDKN1A’s important role in the kidney injury (Zaidan et al., 2020), brain aging (Belsky et al., 2015) and cisplatin cell killing effect (Zamagni et al., 2020). Clinically, it has been shown that cisplatin treatment can lead to kidney injury (Ozkok and Edelstein, 2014) and cognitive impairment (John et al., 2017) in cancer patients.

In addition to cisplatin, we have also found the association between irradiation therapy and aging in 19 out of 46 sample types (GHANDHI BYSTANDER IRRADIATION UP and WARTERS IR RESPONSE 5GY, FDR <0.1, Figure 4A) (Kim et al., 2014). These observations suggested the impact of cisplatin and radiation treatments on aging process in multiple normal tissues.

### 3.3 Drug-Induced Transcriptomic Alterations Recapitulate the Mechanism of Action of the Treatment in L1000 Data

**FIGURE 5:**
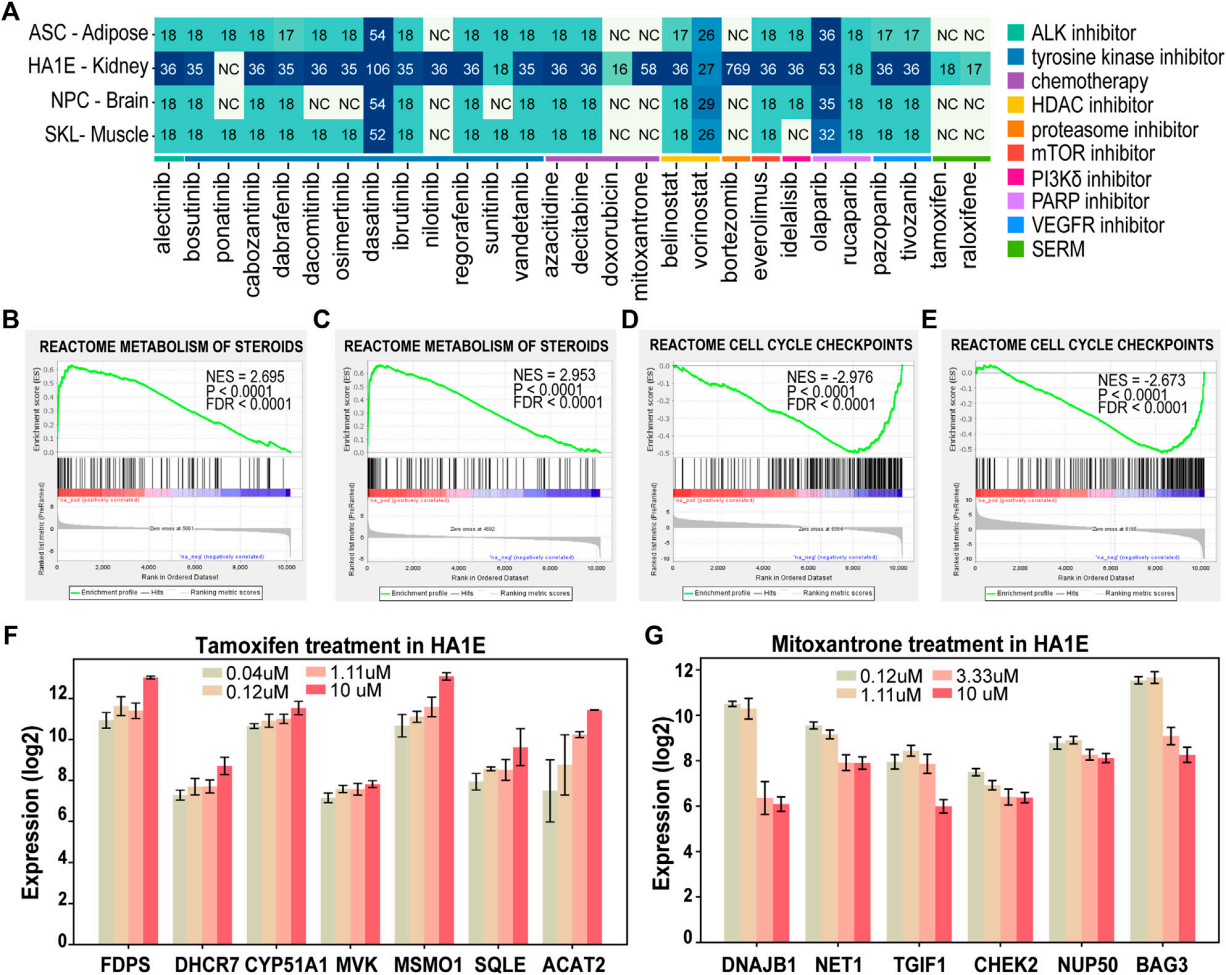
Drug-induced transcriptomic alterations recapitulate the mechanism of action of the treatment in L1000 data. **(A)** The number of expression profiles of normal cells treated by the 28 FDA approved in L1000 data. (NC: Not included, SERM: Selective Estrogen Receptor Modulator). **(B–E)** GSEA analysis of Reactome database gene signatures. **(F)** Genes in “metabolism of steroids” showed increased expression in HA1E cells after tamoxifen treatment at different dosages. **(G)** Genes in “cell cycle checkpoints” showed decreased expression in HA1E cells after mitoxantrone treatment at different dosages. Data are presented as means ± SE.

According to the record of National Cancer Institute, there are 28 FDA approved cancer drugs having enough treatment data points (e.g., dosage and duration) in at least one of four normal/immortalized cell lines in the L1000 data (Figure 5A). Those four cell lines are originated from brain (neural progenitor cells [NPC]), kidney (SV40 + TERT immortalized kidney cell line [HA1E]), muscle (skeletal muscle cells [SKL]), and adipose tissue (adipose tissue-derived mesenchymal stem cells [ASC]).

We first sought to determine if drug-induced transcriptomic alterations can recapitulate the cancer drugs’ known effect on tissue. In this regard, we performed GSEA analyses on drug-induced transcriptomic data of the 28 FDA approved drugs (Liberzon et al., 2015). These analyses revealed that the drug-induced transcriptomic alterations are consistent with the known effect in 22 out 28 FDA drugs (Supplementary Table S1). For example, steroid biosynthesis pathway (METABOLISM OF STEROIDS) was enriched with upregulated genes after treated with selective estrogen receptor modulators (SERMs) such as tamoxifen and raloxifene in HA1E cells (FDR< 0.0001, Figures 5B,C). SERMs are a group of non-steroidal drugs with the ability to bind to estrogen receptors and can upregulate steroid metabolism-related genes by interacting with sterol regulatory element-binding protein (SREBP-2) (Fernández-Suárez et al., 2021). Moreover, important enzymes involved in the “METABOLISM OF STEROIDS” pathway such as farnesyl diphosphate synthetase (FDPS), 7-dehydrocholesterol reductase (DHCR7), methylsterol monooxygenase1 (MSMO1) were upregulated by tamoxifen treatment in a dose-dependent manner (Figure 5F).

For chemotherapy drugs such as mitoxantrone and doxorubicin, cell cycle related pathway “CELL CYCLE CHECKPOINTS” was significantly downregulated after these two treatments (FDR<0.0001, Figures 5D,E). Mitoxantrone are known to work against cancer by killing fast-growing cells. Consistent with its mechanism of, cell cycle regulatory genes such as neuroepithelial cell transforming 1 (NET1), TGFB induced factor homeobox 1 (TGIF1), and checkpoint kinase 2 (CHEK2) were downregulated by mitoxantrone treatment in a dose-dependent manner (Figure 5G). These results suggested the drug-induced transcriptomic alterations and functions are consistent with the mechanisms of action for the treatment.

### 3.4 Interaction Between Cancer Drug-Induced Signatures and Aging-Associated Signatures in Multiple Normal Tissue Types

**FIGURE 6:**
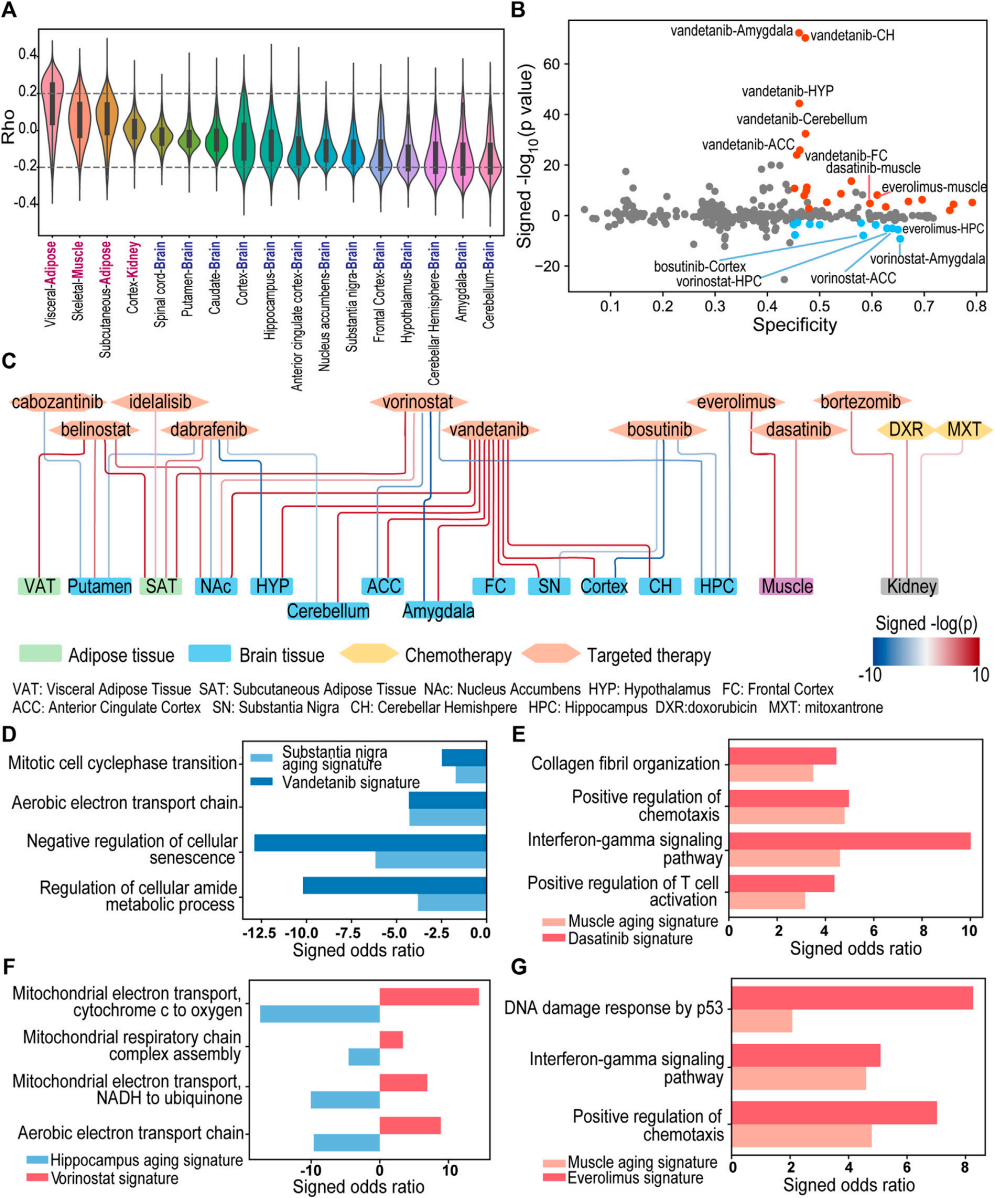
The landscape of drug-aging interaction of 28 FDA approved drugs in brain, kidney, adipose, and muscle. **(A)** The distribution of the correlation between gene expression and the donor’s age in GTEx data. **(B)** The specificity (x-axis) and log-transformed *p*-values of the adjusted concordance ratio (y-axis) for the 326 interactions between drug-induced signatures and aging-associated signatures in multiple normal tissue types. A positive y-value represents CR > 1 and indicates aging-accelerating effect. A negative y-value represents CR < 1 and indicates aging-protective effect. Blue/Red dots are significant interactions with S > 0.45, CR < 0.9/CR > 1.1 and *p* < 0.01. **(C)** Network of 34 significant drug-aging interactions. Blue and red edges indicate protective and accelerating effects respectively. **(D–G)** Pathways enriched with overlapped genes between aging-associated signature and drug-induced signature. A positive/negative Enrichr odds ratio (red/blue bar) represents enrichment with up/downregulated genes.

To determine the drug-aging interaction, we first identified aging-associated signatures in each tissue type in the GTEx dataset and drug-induced signatures in each normal cell line in the L1000 dataset as described in the Section 2. In GTEx database, there are 17 tissue types/regions with sufficient samples (ranging from 85 to 803) that match the 4 normal cell lines tissue origins in the L1000 data (Supplementary Figure S1A). The distribution of the correlation between gene expression and the donor’s age was demonstrated in each tissue type (Figure 6A). Then the number of selected genes varies at different rho thresholds (Supplementary Figure S1B). A reasonable number of genes were selected at rho>0.2 in the aging-associated signatures in different tissues. Interestingly, the total number of signature genes and the percentage of positively/negatively correlated genes are different in each tissue type (Supplementary Figure S1C). For example, a large proportion of signature genes are downregulated in some of the brain tissues of the aged donors while more genes are upregulated in adipose and muscle tissues of the aged donors. This difference might be affected by different sample sizes, but also indicate tissue-specificity of aging-associated signature. Meanwhile, we have observed that the different number of genes whose expression are altered by different drugs, which may be caused by the distinct mechanism of actions (MOA) of the drugs (Supplementary Table S3). Chemotherapy drugs such as doxorubicin and mitoxantrone tend to induce an extensive transcriptomic change in the cell, while targeted therapy drugs may induce less changes in the gene expression.

For each drug-induced signature and aging-associated signature, we calculated a specificity (S) score and an adjusted concordance ratio (CR) to quantify the signature interaction and evaluate the drug-induced aging effects (Figure 1 and see details in Section 2). The drug-aging interaction with a higher specificity score indicates that a drug-induced signature has more overlapped genes with an aging-associated signatures in matched tissue type. On the other hand, the significant adjusted concordance ratio indicates the drug’s accelerating (i.e., CR > 1) or protective (i.e., CR < 1) effect on the aging trajectory of corresponding tissue.

There are 326 pairs of drug-induced signature and aging-induced signature with matched cell tissue origins between GTEx and L1000 datasets. 34% of the drug-aging interactions have a specificity score larger than 0.45 (Figure 6B). For example, chemotherapy drug mitoxantrone and doxorubicin showed high aging specificity scores at 0.749 and 0.756 in kidney. Meanwhile, 36% of the drug-aging interactions have a significant adjusted concordance ratio with *p* < 0.01, including highly significant concordance ratios between vandetanib and aging of multiple brain regions (Figure 6B).

Using a threshold of specificity>0.45, CR < 0.9 or >1.1 and concordance *p* < 0.01, we characterized 34 potential drug-regulated aging events in 15 tissue types/regions (Figure 6C; Supplementary Table S2). Among them, ten drugs were predicted to have 22 events of aging-accelerating effects in 14 tissue types/regions. In particular, doxorubicin was predicted to have aging-accelerating effect in kidney aging with specificity at 0.756 and significantly high adjusted concordance ratio at 1.4 (*p* < 10^−4^), which is consistent with doxorubicin’s established side effect in kidney damage and aging (Su et al., 2015; Yemm et al., 2018). In addition to the aging accelerating effect, our analyses also identified five drugs showing 12 aging protective events in 9 tissue types/regions.

#### 3.4.1 Drug-Aging Interaction Analysis Revealed That Vandetanib (Caprelsa^®^) Treatment May Accelerate Brain Aging

Vandetanib (Caprelsa^®^) is a tyrosine-kinase inhibitor (TKI) of vascular endothelial growth factor receptor (VEGFR), epidermal growth factor receptor (EGFR), and RET tyrosine kinase (LoRusso and Eder, 2008). It has been approved by FDA to treat medullary thyroid cancer. In our study, vandetanib treatment in NPC cells induced gene expression changes demonstrating specificity scores higher than 0.45 and significantly high adjusted concordance ratios with aging-associated signatures in multiple brain regions including amygdala, anterior cingulate cortex, cerebellar hemisphere, cerebellum, cortex, frontal cortex, hypothalamus, nucleus accumbens, and substantia nigra (CR > 1.49, *p* < 0.01, Figure 6C; Supplementary Table S2). Pathways such as “Mitotic cell cycle phase transition” (Enrichr *p*-value <0.05), “Negative regulation of cellular senescence” (Enrichr *p*-value <0.05) and “Regulation of cellular amide metabolic process” (Enrichr *p*-value <0.001) were significantly enriched with the genes that are downregulated in both vandetanib-treated NPC cells and aged substantia nigra tissues (Figure 6D). As a drug that can penetrate the blood-brain barrier (Subbiah et al., 2015), vandetanib treatment may cause dysregulation of cell senescence, mitotic cell cycle, amide metabolism in brain tissue and accelerate aging process of brain. Consistent with our discovery, a recent study has demonstrated that vandetanib exert a deleterious effect on the dopaminergic system in a Parkinson’s disease model (Requejo et al., 2018).

#### 3.4.2 Drug-Aging Interaction Analysis Revealed That Dasatinib (Sprycel®) Treatment May Accelerate Muscle Aging

Our analysis has identified another tyrosine-kinase inhibitor, dasatinib, tend to trigger aging process in muscle tissue. Dasatinib (Sprycel®) was approved to treat Philadelphia chromosome-positive (Ph+) chronic myeloid leukemia and acute lymphoblastic leukemia for both children and adult patients (Keam, 2008). The dasatinib-induced signature in SKL cells has a high specificity score (S = 0.596) and significantly high adjusted concordance ratio (CR = 2.21, *p* < 10^−4^) with aging-associated signature in muscle tissues. Moreover, the overlapped genes between two signatures were significantly enriched in the interferon gamma mediated signaling pathway (Enrichr *p* < 0.001) and alpha/beta T cell activation (Enrichr *p* < 0.05). These results suggest dasatinib may trigger inflammatory response which is observed over-expressed in muscle tissue of aged donors (Figure 6E). Indeed, Naif I AlJohani and co-authors (AlJohani et al., 2015) reported a case of inflammatory myopathy in a patient with chronic myeloid leukemia after treated with dasatinib. There was also a clinical study demonstrating that chronic myeloid leukemia patients on TKI therapy showed significantly more muscle fatigue than control groups (Janssen et al., 2019).

#### 3.4.3 Drug-Aging Interaction Analysis Revealed That Vorinostat (Zolinza®) and Bosutinib (Bosulif®) Treatment May Exert Protective Effect of Brain Aging

The histone deacetylase (HDAC) inhibitor, vorinostat (Zolinza®), was identified to provide protective effect of brain aging. Vorinostat is a FDA approved drug for cutaneous T cell lymphoma treatment (Iwamoto et al., 2013). Vorinostat-induced signature in NPC cells had overall high specificity scores, but significantly low adjusted concordance ratios with aging-associated signatures in multiple brain tissues (hippocampus: S = 0.629, CR = 0.744, *p* < 10^−5^; anterior cingulate cortex: S = 0.639, CR = 0.768, *p* < 10^−5^; amygdala: S = 0.654, CR = 0.83, *p* < 10^−9^). Overlapped genes between aging-associated signature and vorinostat-induced signature were significantly enriched in mitochondrial metabolism pathways such as aerobic respiration (Enrichr *p* < 0.05) (Figure 6F). Our results suggest that vorinostat treatment may reverse mitochondrial dysfunction in brain tissue, which is among the main factors involved in neurodegeneration. Interestingly, *in vitro* study by Surabhi Shukla et al. (2016) showed that vorinostat can independently induce neuritogenesis, and vorinostat treatment can confer memory reinstatement in several cognitive decline mouse models (Kilgore et al., 2010; Benito et al., 2015). Moreover, a phase I clinical trial (NCT03056495) is underway to evaluate the neuroprotective effect of vorinostat in patients with mild Alzheimer disease (VostatAD01, 2017), which supports our discovery of the interactions between vorinostat and brain aging.

Another drug showed protective effect of brain aging is bosutinib (Bosulif®). It is a second generation tyrosine-kinase inhibitor for Bcr-Abl and Src family kinases, which is used for the treatment of chronic myeloid leukemia (Isfort and Brümmendorf, 2018). Bosutinib-induced signature in NPC cells had an overall high specificity, but significantly low concordance ratio with aging-associated signatures in multiple brain regions (hippocampus: S = 0.607, CR = 0.647, *p* < 10^−3^; cortex: S = 0.583, CR = 0.589, *p* < 10^−7^; substantia nigra: S = 0.579, CR = 0.632, *p* < 10^−2^). Bosutinib’s predicted aging protective effect of brain is supported by previous and ongoing studies. In preclinical studies, bosutinib was reported to facilitate the clearance of α-synuclein in Parkinson’s disease (Hebron et al., 2014). Bosutinib treatment also enhanced the clearance of neurotoxic proteins α-amyloid and tau, leading to cognitive improvement in Alzheimer’s disease mouse models (Lonskaya et al., 2013a; Lonskaya et al., 2013b; Hebron et al., 2018). In 2016, a phase I clinical trial (NCT02921477) began to evaluate bosutinib’s effect on patients with mild cognitive impairment and the results support an overall positive outcome after 1 year treatment of bosutinib (National Library of Medicine, 2016; Mahdavi et al., 2021). In 2019, a phase II trial (NCT03888222) was started to test bosutinib as a possible treatment in dementia with Lewy bodies (National Library of Medicine, 2019).

#### 3.4.4 Drug-Aging Interaction Analysis Revealed That Everolimus (Afinitor®) Treatment Demonstrated Aging Protective Effect in Brain but Aging Accelerating Effect in Muscle

The mTOR inhibitor everolimus (Afinitor®) was used to treat multiple cancers (Motzer et al., 2010; Baselga et al., 2011; Yao et al., 2011) and to suppress immunity to prevent rejection in patients having organ transplantation (Tedesco-Silva et al., 2022). Our interaction results showed different effects of the same drug on aging of brain and muscle tissues. Everolimus-induced signature in SKL cells showed aging-accelerating effect in muscle tissue (S = 0.610, CR = 2.765, *p* < 10^−8^). The overlapped positive genes between the two signatures were enriched in functions “DNA damage response by p53,” “interferon-gamma mediated signaling pathway” and “positive regulation of chemotaxis” (Enrich *p* < 0.05, Figure 6G). Consistent with our prediction, previous studies reported that muscle wasting can be induced by everolimus treatment in cancer patients (Albiges et al., 2011; Gyawali et al., 2016). Interestingly, the interaction between everolimus-induced signature and aging-associated signature in brain hippocampus demonstrated aging-protective effect (S = 0.650, CR = 0.424, *p* < 10^−5^). *In vivo* studies from multiple research groups showed that infusion of everolimus restored cognitive function in Alzheimer’s disease models (Fanoudi et al., 2018; Cassano et al., 2019). As a cancer drug that is able to penetrate the blood-brain barrier (Subbiah et al., 2015), everolimus’s potential mechanism in cognitive protection is the protection of intact blood-brain barrier and limited entry of proinflammatory and neurotoxic factors into the brain tissue (Van Skike et al., 2018; Van Skike and Galvan, 2018).

## 4 Discussion

Although laboratory and clinical evidence has shown that several cancer drugs can change the cancer patient aging trajectory, for most of the FDA approved cancer drugs, whether and how they influence the aging process of cancer patient is unknown. More importantly, the molecular and cellular changes underlying the interaction of cancer treatment and altered aging trajectory remain elusive. In this study, we have developed a computational framework to integrate the drug post-treatment transcriptomic data and human tissue transcriptomics data to investigate the drug-induced aging processes in multiple human organs. This strategy allows us to first identify aging-associated signatures in normal aging tissues. We then link the drug treatment effect with aging by comparing the aging-associated signatures to the drug-induced expression signatures using specificity scores and adjusted concordance ratios. By applying this computational framework to L1000 and GTEx databases, we have successfully identified experimentally validated drug-induced effects on aging such as aging-accelerating effects of vandetanib and dasatinib on brain and muscle, respectively. Our result also identified aging-protective effect of vorinostat, everolimus, and bosutinib on brain. With the availability of new data from L1000, GTEx and other databases, our computational strategies can be extended to future analysis of more drugs and tissues.

Our study is an important complement to the clinical and preclinical study. The results from our study can serve as an initial screen of cancer treatments that are likely to induce aging-related consequences in patients. This will provide potential candidate drugs for long term clinical and preclinical study. Moreover, our study can also identify drugs that provide protective effects to tissue aging, which could be considered as candidate drugs that could treat drug-induced side effect of aging or co-treatments to prevent possible aging process. In the future, we will be interested to further utilize this framework to characterize the drugs’ synergistic effect on aging for combination therapy.

Another advantage of our study is that our analysis can help characterize the underlying molecular changes during the drug-aging interaction. For example, Requejo and collogues have used rat model to demonstrate that vandetanib treatment significantly increased behavioral impairment in 6-hydroxydopamine induced preclinical model of Parkinson’s Disease (Requejo et al., 2018). They also observed morphological changes in brain after vandetanib treatment including the decrease of TH-immunopositive striatal volume and the decreased axodendritic network in the substantia nigra. However, the mechanism of how this tyrosine kinase inhibitor influences the dopaminergic system is unknown. In this study, our analyses not only recapitulate the deleterious effect of vandetanib on Parkinson’s Disease, but also reveal that vandetanib treatment causes dysregulation of cell senescence, mitotic cell cycle, amide metabolism in NPC cells. These cellular processes were significantly downregulated in aged substantia nigra human tissues. These observations, combined with the published preclinical animal model result, suggest that vandetanib-mediated cell senescence and metabolism disruption in substantia nigra region may be the mechanism of vandetanib’s deleterious effect on Parkinson’s Disease. By providing the underlying molecular and cellular changes of the drug-induced aging process, our study can support future mechanistic studies and the development of therapeutic strategies to mitigate the drug-induced aging process.

Our study has limitations. One limitation of our study is that it is hard to evaluate the dosage effect of each cancer drug on the aging process in patients. Our drug-induced expression signatures are learned from *in vitro* cell line assays of the L1000 database. The dosages used to treat the normal cell lines in our analysis not necessarily represent the drug concentration in the patient’s normal tissues (e.g., brain) during cancer treatment. In the future study, we will consider the pharmacokinetics and drug tissue distribution (e.g., blood-brain barrier) data to adjust for the practical drug concentration that a cancer patient’s uptake in different tissues. Another limitation of our study is that we established the aging signature in different tissues assuming the tissues are from “healthy” people undergoing natural aging process. However, in reality, aging patients usually have comorbidities such as diabetes or dementia which may influence drugs’ effect on aging. In the future study, we will construct aging signatures with different disease conditions if we can obtain the comorbidities information from the patients. We will build a multivariable regression model that includes the comorbidities as confounding factors to learn the interaction among drugs, aging, and comorbidities.

## Data Availability

The original contributions presented in the study are included in the article/Supplementary Material, further inquiries can be directed to the corresponding author.

## References

[B1] Albiges L. Antoun S. Martin L. Merad M. Loriot Y. Baracos V. (2011). Effect of Everolimus Therapy on Skeletal Muscle Wasting in Patients with Metastatic Renal Cell Carcinoma (mRCC): Results from a Placebo-Controlled Study. J. Clin. Oncol. 29 (7_Suppl. l), 319. 10.1200/jco.2011.29.7_suppl.319

[B2] Alfano C. M. Peng J. Andridge R. R. Lindgren M. E. Povoski S. P. Lipari A. M. (2017). Inflammatory Cytokines and Comorbidity Development in Breast Cancer Survivors Versus Noncancer Controls: Evidence for Accelerated Aging? J. Clin. Oncol. 35 (2), 149–156. 10.1200/JCO.2016.67.1883 27893337 PMC5455675

[B3] AlJohani N. I. Carette S. Lipton J. H. (2015). Inclusion Body Myositis in a Patient with Chronic Myeloid Leukemia Treated with Dasatinib: a Case Report. J. Med. Case Rep. 9 (1), 214. 10.1186/s13256-015-0674-9 26376827 PMC4574179

[B4] Amor D. J. Marsh A. P. Storey E. Tankard R. Gillies G. Delatycki M. B. (2016). Heterozygous Mutations in HSD17B4 Cause Juvenile Peroxisomal D-Bifunctional Protein Deficiency. Neurol. Genet. 2 (6), e114. 10.1212/NXG.0000000000000114 27790638 PMC5070413

[B5] Ashburner M. Ball C. A. Blake J. A. Botstein D. Butler H. Cherry J. M. (2000). Gene Ontology: Tool for the Unification of Biology. The Gene Ontology Consortium. Nat. Genet. 25 (1), 25–29. 10.1038/75556 10802651 PMC3037419

[B6] Baselga J. Campone M. Piccart M. Burris H. A. Rugo H. S. Sahmoud T. (2012). Everolimus in Postmenopausal Hormone-Receptor-Positive Advanced Breast Cancer. N. Engl. J. Med. 366 (6), 520–529. 10.1056/NEJMoa1109653 22149876 PMC5705195

[B7] Belsky D. W. Caspi A. Houts R. Cohen H. J. Corcoran D. L. Danese A. (2015). Quantification of Biological Aging in Young Adults. Proc. Natl. Acad. Sci. U. S. A. 112 (30), E4104–E4110. 10.1073/pnas.1506264112 26150497 PMC4522793

[B8] Benayoun B. A. Pollina E. A. Singh P. P. Mahmoudi S. Harel I. Casey K. M. (2019). Remodeling of Epigenome and Transcriptome Landscapes with Aging in Mice Reveals Widespread Induction of Inflammatory Responses. Genome Res. 29 (4), 697–709. 10.1101/gr.240093.118 30858345 PMC6442391

[B9] Benito E. Urbanke H. Ramachandran B. Barth J. Halder R. Awasthi A. (2015). HDAC Inhibitor-dependent Transcriptome and Memory Reinstatement in Cognitive Decline Models. J. Clin. Invest 125 (9), 3572–3584. 10.1172/JCI79942 26280576 PMC4588238

[B10] Cassano T. Magini A. Giovagnoli S. Polchi A. Calcagnini S. Pace L. (2019). Early Intrathecal Infusion of Everolimus Restores Cognitive Function and Mood in a Murine Model of Alzheimer's Disease. Exp. Neurol. 311, 88–105. 10.1016/j.expneurol.2018.09.011 30243986

[B11] Chen E. Y. Tan C. M. Kou Y. Duan Q. Wang Z. Meirelles G. V. (2013). Enrichr: Interactive and Collaborative HTML5 Gene List Enrichment Analysis Tool. BMC Bioinforma. 14 (1), 128. 10.1186/1471-2105-14-128 PMC3637064 23586463

[B12] Chen J. Wu J. Han C. Li Y. Guo Y. Tong X. (2019). A Mutation in the Filamin C Gene Causes Myofibrillar Myopathy with Lower Motor Neuron Syndrome: a Case Report. BMC Neurol. 19 (1), 198. 10.1186/s12883-019-1410-7 31421687 PMC6697925

[B13] Consortium G. T. Laboratory D. A. Enhancing G. g. Fund N. I. H. C. (2017). Coordinating Center -Analysis Working, G., Statistical Methods Groups-Analysis WorkingGenetic Effects on Gene Expression across Human Tissues. Nature 550 (7675), 204–213. 10.1038/nature24277 29022597 PMC5776756

[B14] Du F. Yu Q. Yan S. Hu G. Lue L. F. Walker D. G. (2017). PINK1 Signalling Rescues Amyloid Pathology and Mitochondrial Dysfunction in Alzheimer's Disease. Brain 140 (12), 3233–3251. 10.1093/brain/awx258 29077793 PMC5841141

[B15] Edwards M. G. Anderson R. M. Yuan M. Kendziorski C. M. Weindruch R. Prolla T. A. (2007). Gene Expression Profiling of Aging Reveals Activation of a P53-Mediated Transcriptional Program. BMC Genomics 8, 80. 10.1186/1471-2164-8-80 17381838 PMC1847444

[B16] Fanoudi S. Hosseini M. Alavi M. S. Boroushaki M. T. Hosseini A. Sadeghnia H. R. (2018). Everolimus, a Mammalian Target of Rapamycin Inhibitor, Ameliorated Streptozotocin-Induced Learning and Memory Deficits via Neurochemical Alterations in Male Rats. EXCLI J. 17, 999–1017. 10.17179/excli2018-1626 30564080 PMC6295637

[B17] Fernández-Suárez M. E. Daimiel L. Villa-Turégano G. Pavón M. V. Busto R. Escolà-Gil J. C. (2021). Selective Estrogen Receptor Modulators (SERMs) Affect Cholesterol Homeostasis through the Master Regulators SREBP and LXR. Biomed. Pharmacother. 141, 111871. 10.1016/j.biopha.2021.111871 34225017

[B18] Gene Ontology Consortium C. (2004). The Gene Ontology (GO) Database and Informatics Resource. Nucleic Acids Res. 32 (Suppl. l_1), 258D–261D. 10.1093/nar/gkh036 PMC308770 14681407

[B19] Guida J. L. Agurs-Collins T. Ahles T. A. Campisi J. Dale W. Demark-Wahnefried W. (2021). Strategies to Prevent or Remediate Cancer and Treatment-Related Aging. J. Natl. Cancer Inst. 113 (2), 112–122. 10.1093/jnci/djaa060 32348501 PMC7850536

[B20] Guida J. L. Ahles T. A. Belsky D. Campisi J. Cohen H. J. DeGregori J. (2019). Measuring Aging and Identifying Aging Phenotypes in Cancer Survivors. J. Natl. Cancer Inst. 111 (12), 1245–1254. 10.1093/jnci/djz136 31321426 PMC7962788

[B21] Gyawali B. Shimokata T. Honda K. Kondoh C. Hayashi N. Yoshino Y. (2016). Muscle Wasting Associated with the Long-Term Use of mTOR Inhibitors. Mol. Clin. Oncol. 5 (5), 641–646. 10.3892/mco.2016.1015 27900103 PMC5103886

[B22] Hebron M. L. Javidnia M. Moussa C. E. (2018). Tau Clearance Improves Astrocytic Function and Brain Glutamate-Glutamine Cycle. J. Neurol. Sci. 391, 90–99. 10.1016/j.jns.2018.06.005 30103978

[B23] Hebron M. L. Lonskaya I. Olopade P. Selby S. T. Pagan F. Moussa C. E. (2014). Tyrosine Kinase Inhibition Regulates Early Systemic Immune Changes and Modulates the Neuroimmune Response in α-Synucleinopathy. J. Clin. Cell Immunol. 5, 259. 10.4172/2155-9899.1000259 25635231 PMC4308054

[B24] Hewitt M. Rowland J. H. Yancik R. (2003). Cancer Survivors in the United States: Age, Health, and Disability. J. Gerontol. A Biol. Sci. Med. Sci. 58 (1), 82–91. 10.1093/gerona/58.1.m82 12560417

[B25] Horvath S. Raj K. (2018). DNA Methylation-Based Biomarkers and the Epigenetic Clock Theory of Ageing. Nat. Rev. Genet. 19 (6), 371–384. 10.1038/s41576-018-0004-3 29643443

[B26] Huntoon V. Widrick J. J. Sanchez C. Rosen S. M. Kutchukian C. Cao S. (2018). SPEG-deficient Skeletal Muscles Exhibit Abnormal Triad and Defective Calcium Handling. Hum. Mol. Genet. 27 (9), 1608–1617. 10.1093/hmg/ddy068 29474540 PMC5905626

[B27] Isfort S. Brümmendorf T. H. (2018). Bosutinib in Chronic Myeloid Leukemia: Patient Selection and Perspectives. J. Blood Med. 9, 43–50. 10.2147/JBM.S129821 29695943 PMC5905837

[B28] Iwamoto M. Friedman E. J. Sandhu P. Agrawal N. G. Rubin E. H. Wagner J. A. (2013). Clinical Pharmacology Profile of Vorinostat, a Histone Deacetylase Inhibitor. Cancer Chemother. Pharmacol. 72 (3), 493–508. 10.1007/s00280-013-2220-z 23820962

[B29] Janssen L. Frambach S. J. C. M. Allard N. A. E. Hopman M. T. E. Schirris T. J. J. Voermans N. C. (2019). Skeletal Muscle Toxicity Associated with Tyrosine Kinase Inhibitor Therapy in Patients with Chronic Myeloid Leukemia. Leukemia 33 (8), 2116–2120. 10.1038/s41375-019-0443-7 30872782 PMC6756217

[B30] John T. Lomeli N. Bota D. A. (2017). Systemic Cisplatin Exposure during Infancy and Adolescence Causes Impaired Cognitive Function in Adulthood. Behav. Brain Res. 319, 200–206. 10.1016/j.bbr.2016.11.013 27851909 PMC5332150

[B31] Kanehisa M. Goto S. (2000). KEGG: Kyoto Encyclopedia of Genes and Genomes. Nucleic Acids Res. 28 (1), 27–30. 10.1093/nar/28.1.27 10592173 PMC102409

[B32] Keam S. J. (2008). Dasatinib. BioDrugs 22 (1), 59–69. 10.2165/00063030-200822010-00007 18215092

[B33] Keating N. L. Nørredam M. Landrum M. B. Huskamp H. A. Meara E. (2005). Physical and Mental Health Status of Older Long-Term Cancer Survivors. J. Am. Geriatr. Soc. 53 (12), 2145–2152. 10.1111/j.1532-5415.2005.00507.x 16398900

[B34] Kerley-Hamilton J. S. Pike A. M. Li N. DiRenzo J. Spinella M. J. (2005). A P53-Dominant Transcriptional Response to Cisplatin in Testicular Germ Cell Tumor-Derived Human Embryonal Carcinoma. Oncogene 24 (40), 6090–6100. 10.1038/sj.onc.1208755 15940259

[B35] Kilgore M. Miller C. A. Fass D. M. Hennig K. M. Haggarty S. J. Sweatt J. D. (2010). Inhibitors of Class 1 Histone Deacetylases Reverse Contextual Memory Deficits in a Mouse Model of Alzheimer's Disease. Neuropsychopharmacology 35 (4), 870–880. 10.1038/npp.2009.197 20010553 PMC3055373

[B36] Kim J. H. Jenrow K. A. Brown S. L. (2014). Mechanisms of Radiation-Induced Normal Tissue Toxicity and Implications for Future Clinical Trials. Radiat. Oncol. J. 32 (3), 103–115. 10.3857/roj.2014.32.3.103 25324981 PMC4194292

[B37] Kitagishi Y. Nakano N. Ogino M. Ichimura M. Minami A. Matsuda S. (2017). PINK1 Signaling in Mitochondrial Homeostasis and in Aging (Review). Int. J. Mol. Med. 39 (1), 3–8. 10.3892/ijmm.2016.2827 27959386

[B38] Landau Y. E. Heimer G. Barel O. Shalva N. Marek-Yagel D. Veber A. (2020). Four Patients with D-Bifunctional Protein (DBP) Deficiency: Expanding the Phenotypic Spectrum of a Highly Variable Disease. Mol. Genet. Metab. Rep. 25, 100631. 10.1016/j.ymgmr.2020.100631 32904102 PMC7451421

[B39] Liberzon A. Birger C. Thorvaldsdóttir H. Ghandi M. Mesirov J. P. Tamayo P. (2015). The Molecular Signatures Database (MSigDB) Hallmark Gene Set Collection. Cell Syst. 1 (6), 417–425. 10.1016/j.cels.2015.12.004 26771021 PMC4707969

[B40] Lonskaya I. Hebron M. L. Desforges N. M. Franjie A. Moussa C. E. (2013a). Tyrosine Kinase Inhibition Increases Functional Parkin-Beclin-1 Interaction and Enhances Amyloid Clearance and Cognitive Performance. EMBO Mol. Med. 5 (8), 1247–1262. 10.1002/emmm.201302771 23737459 PMC3944464

[B41] Lonskaya I. Hebron M. L. Selby S. T. Turner R. S. Moussa C. E. (2013b). Nilotinib and Bosutinib Modulate Pre-plaque Alterations of Blood Immune Markers and Neuro-Inflammation in Alzheimer's Disease Models. Neuroscience 304 (1873-7544), 316–327. (Electronic)). 10.1016/j.neuroscience.2015.07.070 26235435

[B42] López-Otín C. Blasco M. A. Partridge L. Serrano M. Kroemer G. (2013). The Hallmarks of Aging. Cell 153 (6), 1194–1217. 10.1016/j.cell.2013.05.039 23746838 PMC3836174

[B43] LoRusso P. M. Eder J. P. (2008). Therapeutic Potential of Novel Selective-Spectrum Kinase Inhibitors in Oncology. Expert Opin. Investig. Drugs 17 (7), 1013–1028. 10.1517/13543784.17.7.1013 18549338

[B44] Ma S. Avanesov A. S. Porter E. Lee B. C. Mariotti M. Zemskaya N. (2018). Comparative Transcriptomics across 14 Drosophila Species Reveals Signatures of Longevity. Aging Cell 17 (4), e12740. 10.1111/acel.12740 29671950 PMC6052463

[B45] Mahdavi K. D. Jordan S. E. Barrows H. R. Pravdic M. Habelhah B. Evans N. E. (2021). Treatment of Dementia With Bosutinib: An Open-Label Study of a Tyrosine Kinase Inhibitor. Neurol. Clin. Pract. 11, 2163e294–0402e302. (Print)). 10.1212/cpj.0000000000000918 PMC8382351 34484904

[B46] Martín-Maestro P. Gargini R. Perry G. Avila J. García-Escudero V. (2016). PARK2 Enhancement Is Able to Compensate Mitophagy Alterations Found in Sporadic Alzheimer's Disease. Hum. Mol. Genet. 25 (4), 792–806. 10.1093/hmg/ddv616 26721933 PMC4743695

[B47] Motzer R. J. Escudier B. Oudard S. Hutson T. E. Porta C. Bracarda S. (2010). Phase 3 Trial of Everolimus for Metastatic Renal Cell Carcinoma : Final Results and Analysis of Prognostic Factors. Cancer 116 (18), 4256–4265. 10.1002/cncr.25219 20549832

[B48] National Library of Medicine (2016). Study for the Use of TKIs for Treatment of Cognitive Decline Due to Degenerative Dementias. Available at: https://clinicaltrials.gov/ct2/show/NCT02921477 .

[B49] National Library of Medicine (2019). Impact of Bosutinib on Safety, Tolerability, Biomarkers and Clinical Outcomes in Dementia With Lewy Bodies. Available at: https://www.clinicaltrials.gov/ct2/show/NCT03888222 .

[B50] Ozkok A. Edelstein C. L. (2014). Pathophysiology of Cisplatin-Induced Acute Kidney Injury. Biomed. Res. Int. 2014, 967826. 10.1155/2014/967826 25165721 PMC4140112

[B51] Patel S. Barkovich A. J. (2002). Analysis and Classification of Cerebellar Malformations. AJNR Am. J. Neuroradiol. 23 (7), 1074–1087. 12169461 PMC8185716

[B52] Peters M. J. Joehanes R. Pilling L. C. Schurmann C. Conneely K. N. Powell J. (2015). The Transcriptional Landscape of Age in Human Peripheral Blood. Nat. Commun. 6, 8570. 10.1038/ncomms9570 26490707 PMC4639797

[B53] Poretti A. Boltshauser E. (2015). Terminology in Morphological Anomalies of the Cerebellum Does Matter. Cerebellum Ataxias 2, 8. 10.1186/s40673-015-0027-x 26331051 PMC4552363

[B54] Requejo C. Ruiz-Ortega J. A. Bengoetxea H. Bulnes S. Ugedo L. Lafuente J. V. (2018). Deleterious Effects of VEGFR2 and RET Inhibition in a Preclinical Model of Parkinson's Disease. Mol. Neurobiol. 55 (1), 201–212. 10.1007/s12035-017-0733-x 28840516

[B55] Schober P. Boer C. Schwarte L. A. (2018). Correlation Coefficients: Appropriate Use and Interpretation. Anesth. Analg. 126 (5), 1763–1768. 10.1213/ANE.0000000000002864 29481436

[B56] Shukla S. Shariat-Madar Z. Walker L. A. Tekwani B. L. (2016). Mechanism for Neurotropic Action of Vorinostat, a pan Histone Deacetylase Inhibitor. Mol. Cell Neurosci. 77, 11–20. 10.1016/j.mcn.2016.09.003 27678157

[B57] Stathias V. Jermakowicz A. M. Maloof M. E. Forlin M. Walters W. Suter R. K. (2018). Drug and Disease Signature Integration Identifies Synergistic Combinations in Glioblastoma. Nat. Commun. 9 (1), 5315. 10.1038/s41467-018-07659-z 30552330 PMC6294341

[B58] Su Z. Ye J. Qin Z. Ding X. (2015). Protective Effects of Madecassoside against Doxorubicin Induced Nephrotoxicity *In Vivo* and *In Vitro* . Sci. Rep. 5 (1), 18314. 10.1038/srep18314 26658818 PMC4677317

[B59] Subbiah V. Berry J. Roxas M. Guha-Thakurta N. Subbiah I. M. Ali S. M. (2015). Systemic and CNS Activity of the RET Inhibitor Vandetanib Combined with the mTOR Inhibitor Everolimus in KIF5B-RET Re-arranged Non-small Cell Lung Cancer with Brain Metastases. Lung Cancer 89 (1), 76–79. 10.1016/j.lungcan.2015.04.004 25982012 PMC4998046

[B60] Subramanian A. Narayan R. Corsello S. M. Peck D. D. Natoli T. E. Lu X. (2017). A Next Generation Connectivity Map: L1000 Platform and the First 1,000,000 Profiles. Cell 171 (6), 1437–e17. 10.1016/j.cell.2017.10.049 29195078 PMC5990023

[B61] Subramanian A. Tamayo P. Mootha V. K. Mukherjee S. Ebert B. L. Gillette M. A. (2005). Gene Set Enrichment Analysis: a Knowledge-Based Approach for Interpreting Genome-wide Expression Profiles. Proc. Natl. Acad. Sci. U. S. A. 102 (0027-8424), 15545–15550. (Print)). 10.1073/pnas.0506580102 16199517 PMC1239896

[B62] Tedesco-Silva H. Saliba F. Barten M. J. De Simone P. Potena L. Gottlieb J. (2022). An Overview of the Efficacy and Safety of Everolimus in Adult Solid Organ Transplant Recipients. Transpl. Rev. Orl. 36 (1), 100655. 10.1016/j.trre.2021.100655 34696930

[B63] Van Skike C. E. Galvan V. (2018). A Perfect sTORm: The Role of the Mammalian Target of Rapamycin (mTOR) in Cerebrovascular Dysfunction of Alzheimer's Disease: A Mini-Review. Gerontology 64 (3), 205–211. 10.1159/000485381 29320772 PMC5876078

[B64] Van Skike C. E. Jahrling J. B. Olson A. B. Sayre N. L. Hussong S. A. Ungvari Z. (2018). Inhibition of mTOR Protects the Blood-Brain Barrier in Models of Alzheimer's Disease and Vascular Cognitive Impairment. Am. J. Physiol. Heart Circ. Physiol. 314 (4), H693–H703. 10.1152/ajpheart.00570.2017 29351469 PMC5966773

[B65] Verdonschot J. A. J. Vanhoutte E. K. Claes G. R. F. Helderman-van den Enden A. T. J. M. Hoeijmakers J. G. J. Hellebrekers D. M. E. I. (2020). A Mutation Update for the FLNC Gene in Myopathies and Cardiomyopathies. Hum. Mutat. 41 (6), 1091–1111. 10.1002/humu.24004 32112656 PMC7318287

[B66] Violante S. Achetib N. van Roermund C. W. T. Hagen J. Dodatko T. Vaz F. M. (2019). Peroxisomes Can Oxidize Medium- and Long-Chain Fatty Acids through a Pathway Involving ABCD3 and HSD17B4. FASEB J. 33 (3), 4355–4364. 10.1096/fj.201801498R 30540494 PMC6404569

[B67] VostatAD01 (2017). Clinical Trial to Determine Tolerable Dosis of Vorinostat in Patients With Mild Alzheimer Disease (VostatAD01). Available at: https://clinicaltrials.gov/ct2/show/NCT03056495 .

[B68] Wilhelmus M. M. van der Pol S. M. Jansen Q. Witte M. E. van der Valk P. Rozemuller A. J. (2011). Association of Parkinson Disease-Related Protein PINK1 with Alzheimer Disease and Multiple Sclerosis Brain Lesions. Free Radic. Biol. Med. 50 (3), 469–476. 10.1016/j.freeradbiomed.2010.11.033 21145388

[B69] Wu Z. Li S. Tang X. Wang Y. Guo W. Cao G. (2020). Copy Number Amplification of DNA Damage Repair Pathways Potentiates Therapeutic Resistance in Cancer. Theranostics 10 (9), 3939–3951. 10.7150/thno.39341 32226530 PMC7086350

[B70] Yang J. Huang T. Petralia F. Long Q. Zhang B. Argmann C. (2016). Corrigendum: Synchronized Age-Related Gene Expression Changes across Multiple Tissues in Human and the Link to Complex Diseases. Sci. Rep. 6, 19384. 10.1038/srep19384 26795431 PMC4726320

[B71] Yao J. C. Shah M. H. Ito T. Bohas C. L. Wolin E. M. Van Cutsem E. (2011). Everolimus for Advanced Pancreatic Neuroendocrine Tumors. N. Engl. J. Med. 364 (6), 514–523. 10.1056/NEJMoa1009290 21306238 PMC4208619

[B72] Ye X. Sun X. Starovoytov V. Cai Q. (2015). Parkin-mediated Mitophagy in Mutant hAPP Neurons and Alzheimer's Disease Patient Brains. Hum. Mol. Genet. 24 (10), 2938–2951. 10.1093/hmg/ddv056 25678552 PMC4406302

[B73] Yemm K. E. Alwan L. M. Malik A. B. Salazar L. G. (2018). Renal Toxicity with Liposomal Doxorubicin in Metastatic Breast Cancer. J. Oncol. Pharm. Pract. 25 (7), 1738–1742. 10.1177/1078155218798157 30170515

[B74] Zaidan M. Burtin M. Zhang J. D. Blanc T. Barre P. Garbay S. (2020). Signaling Pathways Predisposing to Chronic Kidney Disease Progression. JCI Insight 5 (9). 10.1172/jci.insight.126183 PMC7253021 32376805

[B75] Zamagni A. Pasini A. Pirini F. Ravaioli S. Giordano E. Tesei A. (2020). CDKN1A Upregulation and Cisplatin‑pemetrexed Resistance in Non‑small Cell Lung Cancer Cells. Int. J. Oncol. 56 (6), 1574–1584. 10.3892/ijo.2020.5024 32236605 PMC7170038

