# Supplementary material for: A Computational Framework to Characterize the Cancer Drug Induced Effect on Aging Using Transcriptomic Data
Source: Front Pharmacol. 2022 Jun 29;13:906429. doi: 10.3389/fphar.2022.906429 (PMC9277350; doi:10.3389/fphar.2022.906429)
Supplement: Supplementary file 1 [file Table1.DOCX]

**Table S1. Drug-induced transcriptomic alteration enriched gene sets representing drug mechanism of action**

| **Drug** | **Category** | **Gene Set Name** | **NES**^*^ | **FDR**^†^ |
| --- | --- | --- | --- | --- |
| azacitidine | DNMTi | REACTOME_G2_M_DNA_DAMAGE_CHECKPOINT | 1.90 | 0.069 |
| belinostat | HDAC inhibitor | HELLER_HDAC_TARGETS_UP | 2.40 | <0.001 |
|  |  | PEART_HDAC_PROLIFERATION_CLUSTER_DN | -2.40 | <0.001 |
| bosutinib | bcr-abl inhibitor | KLEIN_TARGETS_OF_BCR_ABL1_FUSION | 1.80 | 0.033 |
| bortezomib | proteasome inhibitor | BIOCARTA_PROTEASOME_PATHWAY | -2.50 | <0.001 |
| cabozantinib | VEGFR inhibitor | WP_VEGFAVEGFR2_SIGNALING_PATHWAY | -1.70 | 0.043 |
| dacomitinib | EGFR inhibitor | KOBAYASHI_EGFR_SIGNALING_24HR_DN | -3.70 | <0.001 |
|  |  | KOBAYASHI_EGFR_SIGNALING_24HR_UP | 1.80 | 0.060 |
| dasatinib | bcr-abl inhibitor | REACTOME_DOWNSTREAM_SIGNALING_EVENTS_OF_B_CELL_RECEPTOR_BCR | -2.30 | <0.001 |
|  |  | REACTOME_SIGNALING_BY_THE_B_CELL_RECEPTOR_BCR | -1.80 | 0.017 |
| decitabine | DNMTi | LIANG_SILENCED_BY_METHYLATION_2 | 2.00 | 0.031 |
| doxorubicin | chemotherapy | REACTOME_CELL_CYCLE_CHECKPOINTS | -2.70 | <0.001 |
| ibrutinib | bcr-abl inhibitor | REACTOME_DOWNSTREAM_SIGNALING_EVENTS_OF_B_CELL_RECEPTOR_BCR | -2.20 | 0.003 |
|  |  | REACTOME_SIGNALING_BY_THE_B_CELL_RECEPTOR_BCR | -1.90 | 0.018 |
| mitoxantrone | chemotherapy | REACTOME_CELL_CYCLE_CHECKPOINTS | -3.00 | <0.001 |
| nilotinib | bcr-abl inhibitor | PID_BCR_5PATHWAY | 1.60 | 0.073 |
| olaparib | PARP inhibitor | SIMBULAN_PARP1_TARGETS_DN | -2.10 | 0.002 |
| osimertinib | EGFR inhibitor | KOBAYASHI_EGFR_SIGNALING_24HR_UP | 2.80 | <0.001 |
|  |  | KOBAYASHI_EGFR_SIGNALING_24HR_DN | -1.80 | 0.034 |
| ponantinib | bcr-abl inhibitor | REACTOME_DOWNSTREAM_SIGNALING_EVENTS_OF_B_CELL_RECEPTOR_BCR | -2.10 | 0.001 |
|  |  | REACTOME_SIGNALING_BY_THE_B_CELL_RECEPTOR_BCR | -1.60 | 0.049 |
| raloxifene | SERM | REACTOME_METABOLISM_OF_STEROIDS | 3.00 | <0.001 |
| regorafenib | VEGFR inhibitor | PID_VEGFR1_PATHWAY | -1.70 | 0.068 |
| sunitinib | VEGFR, PDGFR inhibitor | WP_VEGFAVEGFR2_SIGNALING_PATHWAY | -2.00 | 0.005 |
|  |  | PID_VEGFR1_PATHWAY | -1.60 | 0.042 |
|  |  | WP_TRANSLATION_INHIBITORS_IN_CHRONICALLY_ACTIVATED_PDGFRA_CELLS | -1.50 | 0.065 |
| tamoxifen | SERM | REACTOME_METABOLISM_OF_STEROIDS | 2.70 | <0.001 |
| tivozanib | VEGFR inhibitor | WP_VEGFAVEGFR2_SIGNALING_PATHWAY | -1.70 | 0.046 |
| vandetanib | EGFR inhibitor | KOBAYASHI_EGFR_SIGNALING_24HR_DN | -3.70 | <0.001 |
|  |  | WP_VEGFAVEGFR2_SIGNALING_PATHWAY | -2.10 | 0.001 |
|  |  | KOBAYASHI_EGFR_SIGNALING_24HR_UP | 2.00 | 0.006 |
| vorinostat | HDAC inhibitor | HELLER_HDAC_TARGETS_DN | -2.20 | <0.001 |
|  |  | HELLER_HDAC_TARGETS_UP | 2.50 | <0.001 |

* and † are normalized enrichment score and false discovery rate from the GSEA analyses.
