# Supplementary figures and images for: A Computational Framework to Characterize the Cancer Drug Induced Effect on Aging Using Transcriptomic Data
Source: Front Pharmacol. 2022 Jun 29;13:906429. doi: 10.3389/fphar.2022.906429 (PMC9277350; doi:10.3389/fphar.2022.906429)

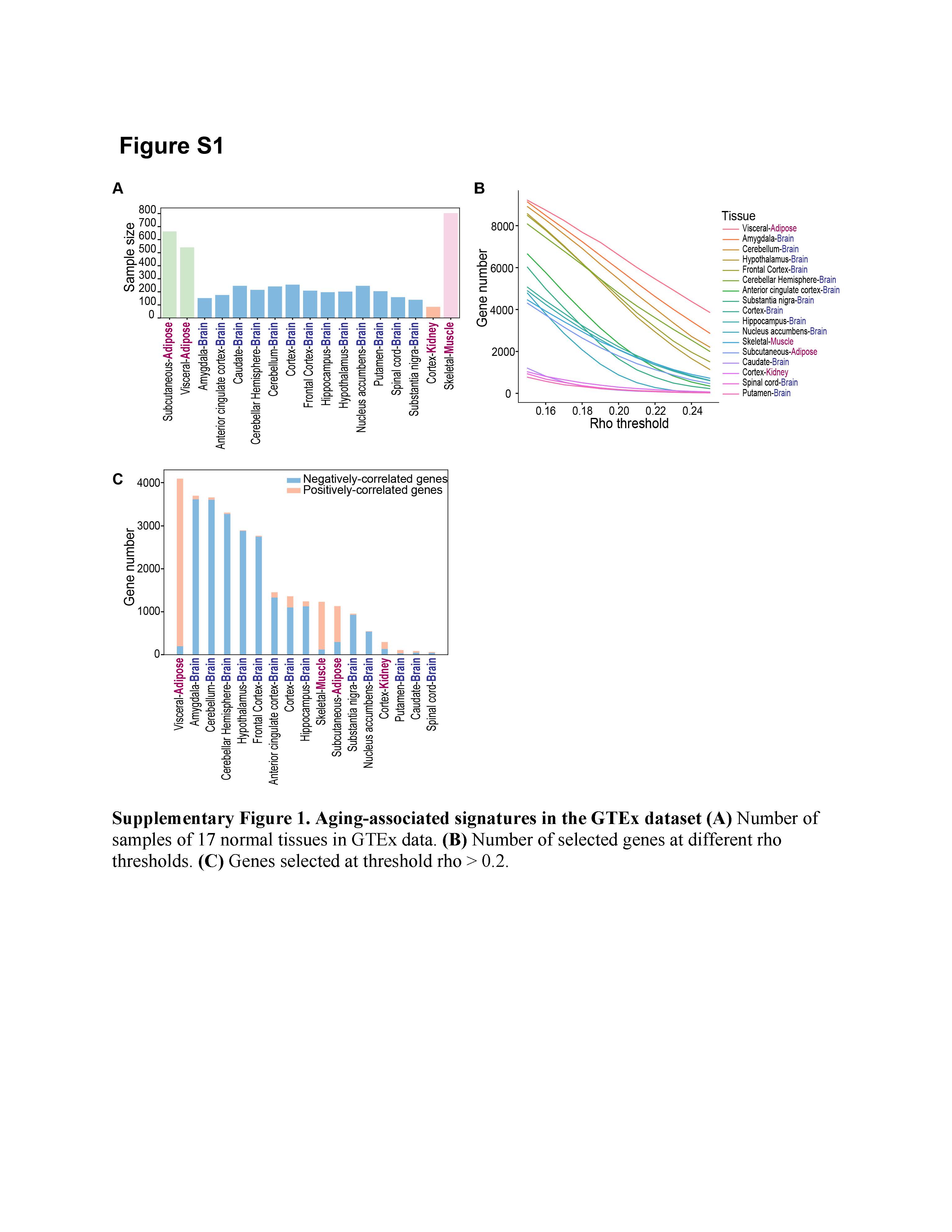

Supplement: Supplementary file 2 [file Image1.JPEG]
