# Supplementary material for: A Computational Framework to Characterize the Cancer Drug Induced Effect on Aging Using Transcriptomic Data
Source: Front Pharmacol. 2022 Jun 29;13:906429. doi: 10.3389/fphar.2022.906429 (PMC9277350; doi:10.3389/fphar.2022.906429)
Supplement: Supplementary file 3 [file Table2.DOCX]

**Table S2.** **The specificity, adjusted concordance ratio and corresponding p-values for the 34 potential drug-regulating aging events identified in the interaction analysis**

| **Drug** | **Tissue** | **Specificity (S)** | **Adjusted Concordance Ratio (CR)** | **CR P value (p)** |
| --- | --- | --- | --- | --- |
| belinostat | Adipose-Visceral | 0.541 | 2.210 | 2.33E-09 |
| belinostat | Adipose-Subcutaneous | 0.561 | 2.514 | 2.30E-14 |
| belinostat | Brain-Nucleus accumbens | 0.696 | 1.403 | 5.27E-07 |
| belinostat | Brain-Putamen | 0.670 | 3.294 | 2.78E-06 |
| bortezomib | Kidney-Cortex | 0.792 | 1.377 | 6.83E-06 |
| bosutinib | Brain-Cortex | 0.583 | 0.589 | 1.25E-08 |
| bosutinib | Brain-Hippocampus | 0.607 | 0.647 | 2.19E-04 |
| bosutinib | Brain-Substantia nigra | 0.579 | 0.632 | 1.14E-03 |
| cabozantinib | Brain-Putamen | 0.500 | 0.310 | 2.40E-04 |
| dabrafenib | Adipose-Subcutaneous | 0.514 | 1.737 | 5.81E-06 |
| dabrafenib | Brain-Cerebellum | 0.457 | 0.803 | 2.00E-03 |
| dabrafenib | Brain-Hypothalamus | 0.453 | 0.696 | 2.05E-08 |
| dabrafenib | Brain-Nucleus accumbens | 0.451 | 0.570 | 2.69E-04 |
| dabrafenib | Brain-Putamen | 0.481 | 0.359 | 3.33E-04 |
| dasatinib | Muscle-Skeletal | 0.596 | 2.214 | 1.58E-05 |
| doxorubicin | Kidney-Cortex | 0.756 | 1.369 | 3.68E-05 |
| everolimus | Muscle-Skeletal | 0.610 | 2.765 | 7.56E-09 |
| everolimus | Brain-Hippocampus | 0.650 | 0.424 | 2.71E-06 |
| idelalisib | Adipose-Subcutaneous | 0.479 | 1.396 | 1.57E-03 |
| mitoxantrone | Kidney-Cortex | 0.749 | 1.222 | 8.89E-03 |
| vandetanib | Brain-Amygdala | 0.461 | 1.903 | 5.02E-73 |
| vandetanib | Brain-Anterior cingulate cortex | 0.462 | 2.073 | 1.36E-26 |
| vandetanib | Cerebellar hemisphere | 0.472 | 1.992 | 4.98E-71 |
| vandetanib | Brain-Cerebellum | 0.473 | 1.488 | 4.26E-33 |
| vandetanib | Brain-Cortex | 0.452 | 1.626 | 1.83E-11 |
| vandetanib | Brain-Frontal cortex | 0.456 | 1.532 | 1.08E-24 |
| vandetanib | Brain-Hypothalamus | 0.461 | 1.791 | 4.25E-45 |
| vandetanib | Brain-Nucleus accumbens | 0.474 | 2.071 | 1.85E-10 |
| vandetanib | Brain-Substantia nigra | 0.475 | 1.764 | 6.51E-12 |
| vorinostat | Adipose-Subcutaneous | 0.470 | 1.825 | 1.27E-08 |
| vorinostat | Brain-Amygdala | 0.654 | 0.830 | 6.14E-10 |
| vorinostat | Brain-Anterior cingulate cortex | 0.639 | 0.768 | 7.93E-06 |
| vorinostat | Brain-Hippocampus | 0.629 | 0.744 | 8.46E-06 |
| vorinostat | Brain-Nucleus accumbens | 0.626 | 1.511 | 3.83E-04 |
