# Supplementary material for: A Computational Framework to Characterize the Cancer Drug Induced Effect on Aging Using Transcriptomic Data
Source: Front Pharmacol. 2022 Jun 29;13:906429. doi: 10.3389/fphar.2022.906429 (PMC9277350; doi:10.3389/fphar.2022.906429)
Supplement: Supplementary file 4 [file Table3.DOCX]

**Table S3. The number of genes included in drug-induced signatures obtained from L1000 dataset.**

| **Drug** | **MOA** | **Cell line** | **Number of Genes** |
| --- | --- | --- | --- |
| dasatinib | tyrosine kinase inhibitor | ASC | 5253 |
| belinostat | HDAC inhibitor | ASC | 5162 |
| cabozantinib | tyrosine kinase inhibitor | ASC | 5141 |
| decitabine | chemotherapy | ASC | 5065 |
| vandetanib | tyrosine kinase inhibitor | ASC | 4986 |
| dabrafenib | tyrosine kinase inhibitor | ASC | 4888 |
| idelalisib | PI3Kδ inhibitor | ASC | 4739 |
| vorinostat | HDAC inhibitor | ASC | 4342 |
| everolimus | mTOR inhibitor | ASC | 4296 |
| pazopanib | VEGFR inhibitor | ASC | 3964 |
| sunitinib | tyrosine kinase inhibitor | ASC | 3951 |
| ibrutinib | tyrosine kinase inhibitor | ASC | 3704 |
| bosutinib | tyrosine kinase inhibitor | ASC | 3619 |
| dacomitinib | tyrosine kinase inhibitor | ASC | 3485 |
| tivozanib | VEGFR inhibitor | ASC | 3277 |
| azacitidine | chemotherapy | ASC | 3230 |
| rucaparib | PARP inhibitor | ASC | 3125 |
| ponatinib | tyrosine kinase inhibitor | ASC | 3109 |
| regorafenib | tyrosine kinase inhibitor | ASC | 3105 |
| osimertinib | tyrosine kinase inhibitor | ASC | 3082 |
| alectinib | ALK inhibitor | ASC | 2979 |
| olaparib | PARP inhibitor | ASC | 1875 |
| doxorubicin | chemotherapy | HA1E | 7235 |
| bortezomib | proteasome inhibitor | HA1E | 7230 |
| mitoxantrone | chemotherapy | HA1E | 6728 |
| belinostat | HDAC inhibitor | HA1E | 5398 |
| vorinostat | HDAC inhibitor | HA1E | 4686 |
| sunitinib | tyrosine kinase inhibitor | HA1E | 4114 |
| dasatinib | tyrosine kinase inhibitor | HA1E | 3283 |
| tamoxifen | SERM | HA1E | 3011 |
| rucaparib | PARP inhibitor | HA1E | 2905 |
| everolimus | mTOR inhibitor | HA1E | 2893 |
| raloxifene | SERM | HA1E | 2856 |
| pazopanib | VEGFR inhibitor | HA1E | 2766 |
| dabrafenib | tyrosine kinase inhibitor | HA1E | 2230 |
| idelalisib | PI3Kδ inhibitor | HA1E | 2032 |
| decitabine | chemotherapy | HA1E | 1847 |
| dacomitinib | tyrosine kinase inhibitor | HA1E | 1820 |
| nilotinib | tyrosine kinase inhibitor | HA1E | 1719 |
| alectinib | ALK inhibitor | HA1E | 1656 |
| osimertinib | tyrosine kinase inhibitor | HA1E | 1642 |
| cabozantinib | tyrosine kinase inhibitor | HA1E | 1488 |
| bosutinib | tyrosine kinase inhibitor | HA1E | 1118 |
| tivozanib | VEGFR inhibitor | HA1E | 1083 |
| ibrutinib | tyrosine kinase inhibitor | HA1E | 1040 |
| vandetanib | tyrosine kinase inhibitor | HA1E | 1024 |
| azacitidine | chemotherapy | HA1E | 813 |
| regorafenib | tyrosine kinase inhibitor | HA1E | 775 |
| olaparib | PARP inhibitor | HA1E | 430 |
| belinostat | HDAC inhibitor | NPC | 6840 |
| vorinostat | HDAC inhibitor | NPC | 6252 |
| everolimus | mTOR inhibitor | NPC | 6035 |
| bosutinib | tyrosine kinase inhibitor | NPC | 5479 |
| azacitidine | chemotherapy | NPC | 4671 |
| vandetanib | tyrosine kinase inhibitor | NPC | 4442 |
| dabrafenib | tyrosine kinase inhibitor | NPC | 4433 |
| decitabine | chemotherapy | NPC | 4009 |
| cabozantinib | tyrosine kinase inhibitor | NPC | 3853 |
| idelalisib | PI3Kδ inhibitor | NPC | 3759 |
| pazopanib | VEGFR inhibitor | NPC | 3479 |
| regorafenib | tyrosine kinase inhibitor | NPC | 3074 |
| alectinib | ALK inhibitor | NPC | 3017 |
| ibrutinib | tyrosine kinase inhibitor | NPC | 3012 |
| rucaparib | PARP inhibitor | NPC | 2913 |
| tivozanib | VEGFR inhibitor | NPC | 2800 |
| dasatinib | tyrosine kinase inhibitor | NPC | 2037 |
| olaparib | PARP inhibitor | NPC | 1349 |
| belinostat | HDAC inhibitor | SKL | 6583 |
| everolimus | mTOR inhibitor | SKL | 6082 |
| dasatinib | tyrosine kinase inhibitor | SKL | 5399 |
| ponatinib | tyrosine kinase inhibitor | SKL | 5376 |
| azacitidine | chemotherapy | SKL | 5173 |
| bosutinib | tyrosine kinase inhibitor | SKL | 4849 |
| vorinostat | HDAC inhibitor | SKL | 4743 |
| cabozantinib | tyrosine kinase inhibitor | SKL | 4557 |
| tivozanib | VEGFR inhibitor | SKL | 4162 |
| dabrafenib | tyrosine kinase inhibitor | SKL | 4161 |
| regorafenib | tyrosine kinase inhibitor | SKL | 3959 |
| vandetanib | tyrosine kinase inhibitor | SKL | 3884 |
| sunitinib | tyrosine kinase inhibitor | SKL | 3541 |
| pazopanib | VEGFR inhibitor | SKL | 3481 |
| rucaparib | PARP inhibitor | SKL | 3371 |
| dacomitinib | tyrosine kinase inhibitor | SKL | 3343 |
| alectinib | ALK inhibitor | SKL | 3163 |
| ibrutinib | tyrosine kinase inhibitor | SKL | 3012 |
| osimertinib | tyrosine kinase inhibitor | SKL | 2930 |
| decitabine | chemotherapy | SKL | 2867 |
| olaparib | PARP inhibitor | SKL | 1340 |
